# Onion-cadmium-renal function: Relationship?

**DOI:** 10.4103/0971-4065.73434

**Published:** 2010-10

**Authors:** V. Wiwanitkit

**Affiliations:** Wiwanitkit House, Bangkhae, Bangkok – 10160, Thailand

Sir,

I read the recent report by Ige *et al*, with a great interest.[1] Ige *et al*, concluded that “Cd exposure causes renal dysfunction, but oral administration of onion could prevent it.”[1] This animal model study is very interesting but further implication for clinical practice in human beings should be carefully done. First, the study on a few subjects in this work might have limitation in identification of toxicity and confirmation of effectiveness of the extract. Second, whether the extract process has any modification in biochemical and physical properties of active herbal gradients in onion is questionable. If there is any modification, the actual clinical use must be the extracted regimen not crude onion. Third, the metabolism of Cd is affected by cytochrome polymorphism.[2] There is no information on this aspect for studied subjects in the report by Ige *et al*.[1] Finally, if onion has actual pharmacological effect for the improvement of renal function, the exact dosage for human beings has to be further assessed.

## References

[1] Ige SF, Salawu EO, Olaleye SB, Adeeyo OA, Badmus J, Adeleke AA (2009). Onion (Allium cepa) extract prevents cadmium induced renal dysfunction. Indian J Nephrol.

[2] Thévenod F (2003). Nephrotoxicity and the proximal tubule: Insights from cadmium. Nephron Physiol.

